# Kinetics of Nitric Oxide and Oxygen Gases on Porous Y-Stabilized ZrO_2_-Based Sensors

**DOI:** 10.3390/molecules18089901

**Published:** 2013-08-16

**Authors:** Sajin Killa, Ling Cui, Erica P. Murray, Daniela S. Mainardi

**Affiliations:** 1 Chemical Engineering and Institute for Micromanufacturing, Louisiana Tech University, Ruston, LA 71272, USA; E-Mail: ski002@latech.edu; 2 Institute for Micromanufacturing, Louisiana Tech University, Ruston, LA 71272, USA; E-Mails: lcu003@latech.edu (L.C.); emurray@latech.edu (E.P.M.)

**Keywords:** NO_x_, diesel emissions, YSZ, NO sensing, porous, density functional theory, reaction mechanism

## Abstract

Using impedance spectroscopy the electrical response of sensors with various porous Y-stabilized ZrO_2_ (YSZ) microstructures was measured for gas concentrations containing 0–100 ppm NO with 10.5%O_2_ at temperatures ranging from 600–700 °C. The impedance response increased substantially as the sensor porosity increased from 46%–50%. Activation energies calculated based on data from the impedance measurements increased in magnitude (97.4–104.9 kJ/mol for 100 ppm NO) with respect to increasing YSZ porosity. Analysis of the oxygen partial pressure dependence of the sensors suggested that dissociative adsorption was the dominant rate limiting. The PWC/DNP theory level was used to investigate the gas-phase energy barrier of the 2NO+O_2_→2NO_2_ reaction on a 56-atom YSZ/Au model cluster using Density Functional Theory and Linear Synchronous Transit/Quadratic Synchronous Transit calculations. The reaction path shows oxygen surface reactions that begin with NO association with adsorbed O_2_ on a Zr surface site, followed by O_2_ dissociative adsorption, atomic oxygen diffusion, and further NO_2_ formation. The free energy barrier was calculated to be 181.7 kJ/mol at PWC/DNP. A qualitative comparison with the extrapolated data at 62% ± 2% porosity representing the YSZ model cluster indicates that the calculated barriers are in reasonable agreement with experiments, especially when the RPBE functional is used.

## 1. Introduction

Nitrogen oxide (NO_x_) exhaust sensors are typically high temperature devices designed to operate in combustion environments. Several automobile companies such as Toyota, Ford and Chrysler have put an effort toward research in developing more efficient NO_x_ sensors, and many academic and government institutions are also involved in developing such sensors [1,2,3,4]. 

Emission gases have been a focus area of study because they consist of major greenhouse gases that cause environmental hazards such as air pollution. Such gases include oxides of carbon (CO and CO_2_), oxides of nitrogen (NO and NO_2_) and oxides of sulphur. These gases produce acid rain, smog and ozone and have adverse effects if they are not controlled. In the United States, the Environmental Protection Agency (EPA) oversees emission standards, which are defined by the Clean Air Act Amendments [5,6]. NO_x_ derivatives are one of the most highly studied exhaust gases which includes NO (nitric oxide, 93%), NO_2_ (nitrogen dioxide, 5%) and other derivatives (2%). The major issue driving the research is the need for more rapid, sensitive, reliable and cost-efficient sensors for these applications. NO_x_ exhaust gas sensors presently available in the market are capable of detecting NO_x_ down to about 10 ppm. Further research is necessary to detect these gases at concentrations approaching 1 ppm.

Ceramic oxide-based electrochemical sensors are widely used as NO_x_ sensors as they can rapidly respond to changes in the exhaust gas composition. Yttria-stabilized zirconia (YSZ) is favored as the electrolyte in these sensors due to its tolerance for both lean and rich exhaust environments. Under lean exhaust conditions, the oxygen concentration in the exhaust is high. By contrast, rich conditions create an oxygen reducing environment. YSZ also has a relatively high oxygen ion conductivity, which contributes to the magnitude of the sensor response [7,8]. 

Typically, the NO_x_ sensor is composed of porous Pt electrodes with a dense YSZ electrolyte. However, recent studies have shown increased NO_x_ sensitivity for sensors fabricated with dense non-catalytic electrodes and a porous YSZ electrolyte [9,10]. The non-catalytic dense electrodes seem to limit heterogeneous catalysis, which ultimately increases NO_x_ sensitivity. For such sensors, the YSZ electrolyte must be porous to allow for gas diffusion. In addition, when non-catalytic electrodes are used, the catalytic activity of the YSZ electrolyte becomes important. Few studies for this novel NO_x_ sensor design have considered the influence of the porous electrolyte on NO_x_ sensing. Catalytic studies by Yang *et al.* confirmed that there are strong molecular interactions between NO_x_ species and YSZ [11]. Other studies have found that modifying the surface area of YSZ can improve the NO_x_ sensor response depending on the operating conditions [12,13]. 

The redox chemical reactions involved in the sensing process are given by Equations (1–3):

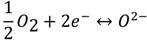
(1)

*NO*_2_ + 2*e*^−^ ↔ *NO* + *O*^2−^ (2)

2*NO* + *O*_2_ ↔ 2*NO*_2_ (3)

According to these redox reactions, NO tends to be partially oxidized to form NO_2_. Reactions given by Equations (1) and (2) are known to occur simultaneously, with reaction rates that depend on the gas phase chemistry and the sensor microstructure. The different modes of operation and variation in microstructures have made it difficult to fully elucidate the role of porous YSZ on gas sensing. Greater understanding of the mechanisms governing YSZ and NO_x_ interactions will contribute to the design and optimization of NO_x_ exhaust gas sensors. 

In this paper, a study on porous YSZ-based NO_x_ sensors with dense gold electrodes is presented, in order to gain greater insight on the mechanisms governing porous YSZ in NO_x_ sensing. To the best of the authors knowledge the combined experimental and modeling work presented here represent a first attempt to analyze the influence of various porous YSZ microstructures on NO_x_ sensing, and investigate the NO and O_2_ gas phase reaction that take place at the molecular level during the sensing process.

## 2. Methodology

### 2.1. Experimental Procedures

#### 2.1.1. Sensor Fabrication

**Figure 1:**
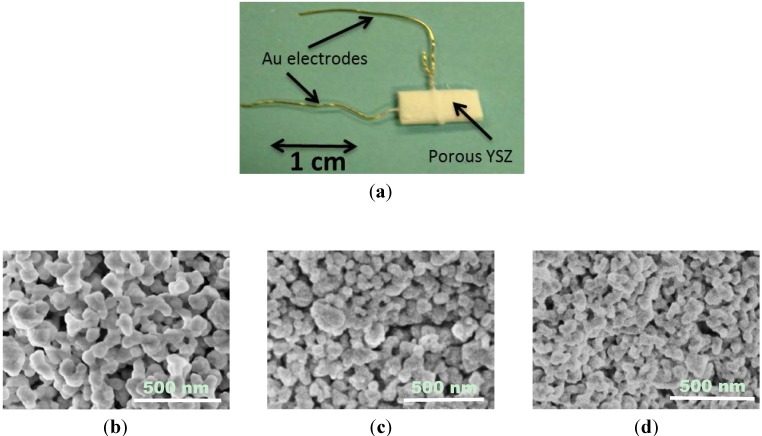
(**a**) NO_x_ exhaust gas sensor. The porous YSZ microstructure for sensors fabricated at (**b**) 950 °C, (**c**) 1000 °C, and (**d**) 1050 °C, where the resulting porosity was 50, 48 and 46%, respectively.

NO_x_ sensors with a porous YSZ (8 mol% Y_2_O_3_-doped ZrO_2_, Tosoh Corp., Grove City, OH, USA) electrolyte and Au wire (0.25 mm diameter, Alfa Aesar, Ward Hill, MA, USA) electrodes were prepared using tape casting and standard ceramic processing methods. The tape formulation consisted of B-98 Butvar (binder), dipropylene glycol benzoate (plastizer), phosphate ester (dispersant), xylene and ethanol (solvents) mixed with the YSZ powder. The mixture was ball milled using a 3D rotational mill to establish a homogeneous slurry. The slurry was cast on silicone coated mylar using the doctor blade technique and allowed to dry in air at room temperature. The YSZ tape was cut into approximately 1 cm × 0.5 cm pieces in order to construct the sensor. For each sensor, a Au wire electrode was placed between 2 layers of YSZ tape. A slurry made by dissolving additional YSZ tape into ethanol was also applied between the 2 layers in order to bond the YSZ tape layers together. Another Au wire was wrapped around the outer layers of YSZ tape, and a coating of the YSZ slurry was applied around the outside of the entire assembly to complete the sensor [Figure 1(a)]. 

The sensors were allowed to dry overnight in air and the final thickness of the sensors was approximately 0.2 cm. To establish three different porous microstructures, the sensors were fired at 950 °C (Sensor A), 1,000 °C (Sensor B), and 1,050 °C (Sensor C) for 1 h. Several sensors were fabricated for each sensor type and the data presented in this work describes the common characteristics and behavior observed for each type of sensor. Scanning electron microscopy (SEM) was used to observe the morphology and microstructure of the sensors. These images indicated pore sizes of approximately 60–80 nm and the porosity was estimated to be 50%, 48%, and 46% for Sensors A, B, and C, respectively [Figure 1(b–d)]. 

#### 2.1.2. Sensor Testing

A Gamry Reference 600 (Gamry Instruments, Warminster, PA, USA) was used to carry out impedance spectroscopy measurements on the various sensors in order to characterize their electrical responses. Impedance spectroscopy is a powerful technique for investigating the dynamics of mobile and bound charges within the bulk or at the interface of solid and liquid materials. The impedance of a sample is obtained by applying a small signal stimulus (voltage or current) across a sample and measuring the electrical response that depends on the frequency of the signal. Analysis of the impedance data can offer insight regarding rate limiting reactions affecting the NO_x_ response of the sensor. 

In this work, impedance experiments were carried out by attaching the sensor to an apparatus that was loaded into a quartz tube and placed in a furnace. The Au wire electrodes from the sensor were connected to the Gamry impedance analyzer. Dry mixtures of N_2_, O_2_, NO, and NO_2_ were introduced to the quartz tube using a standard gas monitoring system equipped with thermal mass flow controllers. Experiments were carried out by flowing 0–100 ppm NO and NO_2_ with 1%–18% O_2_ and a balance of N_2_ through the quartz tube at a total flow rate of 100 cm^3^/min. Measurements were collected at temperatures ranging from 600–700 °C. The Gamry impedance analyzer was configured to apply a signal amplitude of 50 mV and collect measurements over a frequency range of 1–10^6^ Hz at 10 steps per decade. Additional current-voltage measurements were collected to verify that the 50 mV alternating signal was well within the linear current-voltage regime for the sensors. Thus, data analysis based on a linear approximation could be applied with high accuracy.

### 2.2. Computational Procedures

#### 2.2.1. Sensor Model

When Y_2_O_3_ is added to ZrO_2_ during the manufacturing of YSZ, the zirconia cubic fluorite structure stabilizes and transforms the non-conducting zirconia into an ion conducting material. This doping with Y_2_O_3_, creates the substitution of the Zr^4+^ cations in the original ZrO_2_ by the Y^3+^ cations; thereby forming oxygen vacancies to maintain charge neutrality in the resulting YSZ lattice. Even though several experimental and theoretical work have been published regarding the use of YSZ, only incomplete and controversial information regarding its structural properties have been reported. For this reason, a 40-atom model cluster of this material supported on a 16-atom Au cluster representing the Au wire has been chosen for this work to include the known atomistic structural details needed to investigate and test the reaction given by Equation (3). 

The δ-YSZ crystal structure [14] was used to build the YSZ model cluster using the crystal builder option of the Materials Studio^®^ 6.0 software by Accelrys, Inc. (San Diego, CA, USA). This YSZ structure belongs to the *P*1 space group and corresponds to the cubic fluorite structure with molecular formula Y_4_Zr_3_O_12_, and lattice parameters of *a* = 6.458 Å, *b* = 6.652 Å and *c* = 6.415 Å. Then, first-principles calculations were used to investigate the gas-phase internal energy of activation and free energy barrier of the reaction given by Equation (3) in the presence of the 56-atom YSZ/Au model cluster.

#### 2.2.2. Ground State Conformations

The Local Gradient Approximation (LDA) method [15,16] within the Density Functional Theory formalism [15,16], as implemented in the DMOL^3^ module of the Materials Studio^®^ 6.0 software, was used in this work [17]. All geometry optimization calculations were performed using the Perdew-Wang (PWC) functional in combination with the double numerical with polarization (DNP) basis set, since this was the best set available [17] in DMOL^3^. This basis set considers a polarization *d* function on heavy atoms and a polarization *p* function on hydrogen atoms. It compares to the split-valence double zeta 6-31G** in size; however, it is more accurate than the Gaussian basis sets of the same size [18]. 

The use of the PWC/DNP theory level has been proven to be adequate for the study of solid phases, as it can provide a balanced description of the overall structure and energetics of materials, such as porphyrin−fullerene C60 complexes [19,20,21], carbon nanotubes [20], thiophene-based oligomers and polymers [22,23], and molecular systems containing charged particles [24], at a moderate computational cost. When the PWC/DNP theory level is used, errors in energies are expected to be in the order of 8–20 kJ/mol; similar to those expected when using the Becke-3 hybrid functionals in combination with the 6-31G** basis set [15,16,25]. 

Harmonic vibrational frequency calculations were performed to ensure that stationary points on the potential energy surface of the molecular systems are, in fact, local minima (all real frequencies) or transition states (only one imaginary frequency). Spin multiplicity states were also checked and zero point energy corrections considered in all calculations. Additionally, in order to obtain accurate results, diverse possible arrangements of the reactant, intermediates and product pertaining to Equation (3) were investigated, and in some cases, more than one optimized energy configurations along the real potential energy surface of the molecular system were identified. The energies obtained for the different minima were compared, and only the ground state conformations were considered for calculating transition state structures. 

#### 2.2.3. Reaction Kinetics Modeling

Appropriate reactants and products involved in the reaction shown in Equation (3) were considered for defining atom pairing, so that a 3D trajectory file representing the reaction path preview was generated with the Reaction Preview tool of the Materials Studio software. Then, these 3D trajectory files were used as inputs to obtain the corresponding transition states, using the linear synchronous transit and quadratic synchronous transit (LST/QST) calculation with conjugate gradient minimization [26] within the Transition State search tool in DMOL^3^. This methodology starts with a LST/optimization (bracketing the maximum between the reactant and product and performing energy minimization of the obtained maximum in the reaction pathway). The Transition State hence obtained was used as starting point for performing a finer search with the QST/optimization followed by a conjugate gradient minimization. This cycle was repeated until a stationary point with only one imaginary frequency (transition state) was found [26]. When finding more than one negative frequency, the corresponding (imaginary) modes of vibrations are animated in order to visualize the mode that would eventually follow the intended step from the particular reactant to product. That particular mode is then selected to perform the transition state optimization to verify whether the obtained geometry is indeed a transition state.

The transition state finally obtained by the LST/QST/conjugate gradient method may not be the transition state connecting the intended reactant and product for a particular reaction. Therefore, in order to thoroughly investigate the reaction path, the intrinsic reaction coordinate (IRC) analysis was performed. In DMOL^3^, the IRC calculations are included in the Transition State Confirmation tool [17]. This tool starts at the transition state and locates successive minima in the direction of the reactant and product paths. This path is known as the minimum energy path, which should connect the supposed transition state to the presumed reactant and product [27]. It uses the nudged elastic band method to validate a transition state by introducing a fictitious spring force which connects the neighboring points to ensure continuity of the path and then it projects the force, so that the system converges to the minimum energy path [27]. This procedure for exploring the energy path associated to a reaction has been successfully used in other work involving solids [28] and clusters [29,30,31].

The internal energy of activation at absolute zero, ΔU, is a property derived from the Born-Oppenheimer electronic energies and the vibrational zero point energies, which are not affected by temperature; whereas, the so called Arrhenius activation energy, *E_a_*, depends on temperature [32]. Hence, the internal energy of activation ΔU is not the same as the Arrhenius activation energy, *E_a_* [33,34]. In this work, information on the calculated internal energy of activation, ΔU, and the Gibbs Free energy barrier (internal energy of activation with the thermal corrections to the Gibbs Free energy at different temperatures included), ΔG(T), for the forward reaction given by Equation (3) are provided, based on geometry optimization calculations conducted at the LDA PWC/DNP theory level. 

Typically bond energies and the cohesive energies of solid materials are overestimated when LDA functionals are used [35]. These properties and the energy barriers for molecular reactions involving solids can be greatly improved by using Generalized Gradient Approximation (GGA) methods. Hence, in order to provide better kinetic results, single point energy calculations using the DNP basis set on the structures optimized at the PWC/DNP theory level were performed using three Generalized Gradient Approximation (GGA) functionals. The GGA functionals used include the Perdew-Wang-91 functional (PW91) [36], the Perdew-Burke-Ernzerhof (PBE) functional [37], and the revised PBE functional (RPBE). From these three functionals, the RPBE is typically found to be superior in the description of the energetics of atomic and molecular bonding to surfaces [38]. 

## 3. Results and Discussion

### 3.1. Impedance Response

Generally, the impedance response is plotted with respect to the real, Z’, and imaginary, Z”, axes in the complex plane, commonly called a Nyquist plot [39]. The impedance is then expressed as: Z(*ω*) = Z’ + *j*Z”, where the angular frequency is *ω*. For an ideal sample, the impedance is described by a perfect semicircular arc. In practice, it is not uncommon for multiple and distorted arcs to be present in the impedance spectra. Each arc in the spectra relates to at least one rate limiting reaction step. Impedance arcs occurring over the high frequency domain generally indicate that electrochemical reactions (e.g., charge transfer) are rate limiting. Arcs displayed in the low frequency regime suggest surface related process are limiting. Analysis of the activation energy and oxygen partial pressure dependence can help to interpret rate-limiting processes. Correlating the rate limiting process with the microstructure of the sensor is expected to enable optimization of the fabrication process to reduce or eliminate rate-limiting mechanisms and promote NO_x_ sensor performance.

**Figure 2:**
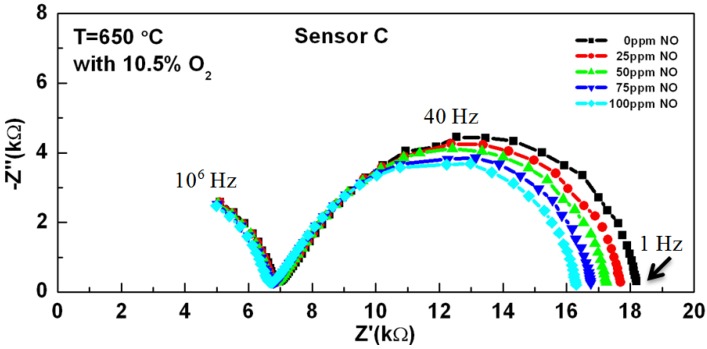
Nyquist plots of the sensors fired at 1,050 °C while operating in 10.5% O_2_ with 0–100 ppm NO present with N_2_ balance.

Typical impedance data describing the electrical response of the sensors to various concentrations of NO are shown in the Nyquist plots in Figure 2. The impedance data for the sensors indicated a partial high frequency arc along with a complete arc at lower frequencies. Higher frequencies beyond the capabilities of the Gamry Reference 600 would be necessary to resolve the complete high frequency arc. For this study, the low frequency arc was most relevant as the electrical response of NO_x_ reactions was described in this regime. Data obtained for NO_2_ (not shown) was nearly identical to that for NO, suggesting equilibrium conditions according to Equation (3) were achieved [13]. At equilibrium for temperatures above 650 °C over 90% NO is present according to thermodynamic calculations. For these reasons the data presented concentrates on the NO response. 

As seen in Figure 2, the sensor response to NO became more significant at frequencies below 40 Hz, as greater distinction was observed between the impedance arcs for NO concentrations between 0–100 ppm. For all of the sensors, the impedance response became smaller as the concentration of NO increased, which was likely related to more molecular reactions taking place. The impedance data for Sensors A and B demonstrated the same trends as shown for the NO response for Sensor C, however, the magnitude of the impedance arcs were different. This was attributed to the different microstructures. Sensor A had the largest porosity (50%), and the largest impedance arcs; whereas, Sensor C had the smallest porosity (46%) and the smallest arcs. 

**Figure 3:**
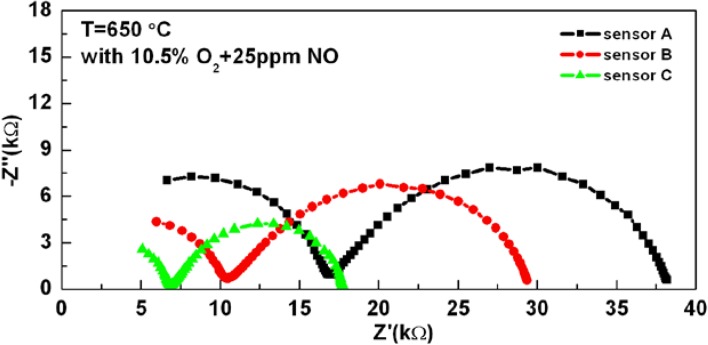
Nyquist plots of the sensors fired at 950 °C (sensor A), 1,000 °C (sensor B), and 1,050 °C (sensor C) while operating in 10.5% O_2_ with 25 ppm NO present with N_2_ balance.

The effect of porosity on the impedance is shown in Figure 3 for sensors A, B, and C when 10.5% O_2_, 25 ppm NO, and N_2_ was present. The magnitude of the impedance response increased with increasing porosity, which was related to oxygen ion transport resistance in YSZ [40]. As the porosity increased, the contact between YSZ particles decreased and subsequently reduced the available pathways for ionic transport. In addition, increasing porosity reduced YSZ contact at the triple phase boundary (TPB), which are locations where the gas, electrode and electrolyte meet. Reactions impacting NO sensing [described by Equations (1) and (2)] are understood to occur along the TPB such that modifications to the density of available TPB sites can promote or hinder the NO response. Additional reactions can occur in the gas phase, such as Equation (3), which applies to surface reactions. The mechanisms governing these reactions are commonly interpreted from analysis of the temperature dependence and oxygen partial pressure data, which is discussed next.

### 3.2. Activation Energy

**Figure 4:**
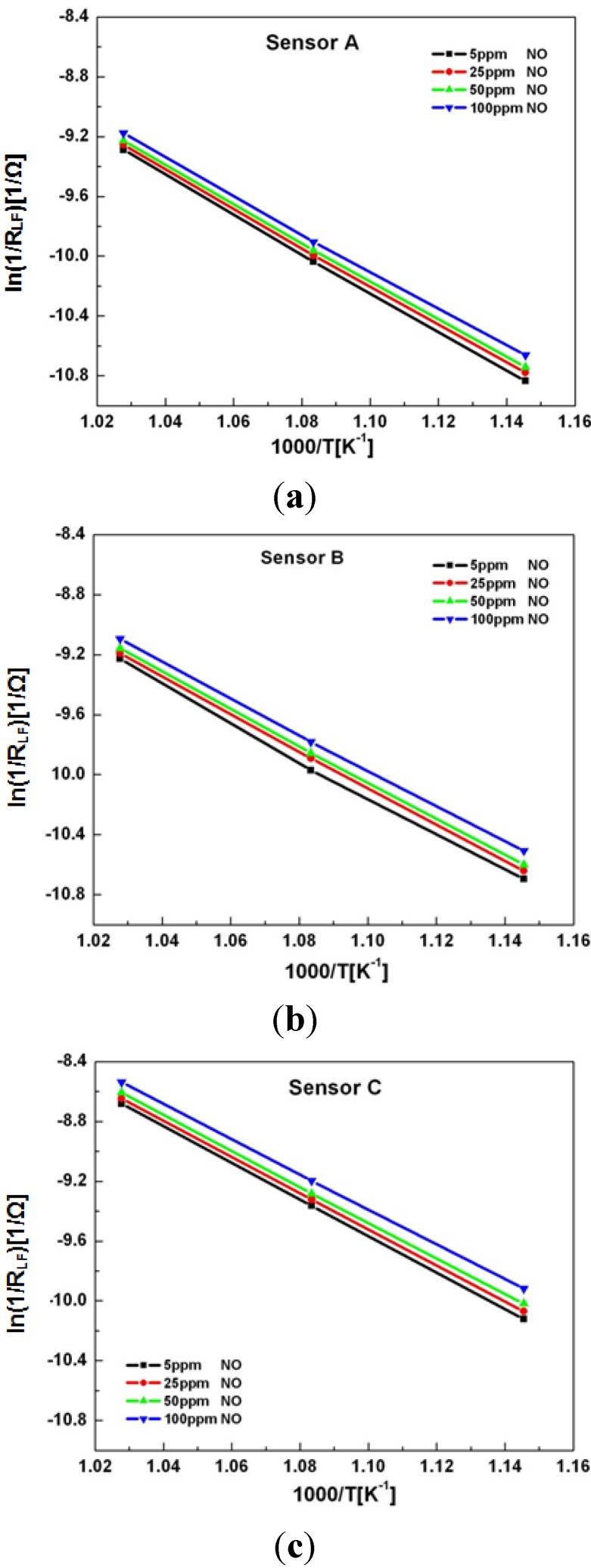
Arrhenius plots for sensors with porosity of: (**a**) 50% (Sensor A), (**b**) 48% (Sensor B), and (**c**) 46% (Sensor C), while operating in 10.5% O_2_ with 5–100 ppm NO present in a balance of N_2_.

The temperature dependence of sensors A, B, and C was based on impedance measurements of the low frequency arc collected at 600 °C, 650 °C and 700 °C. Measurements were collected for various concentrations of NO, and the activation energy, *E_a_* based on experimental data was determined from the slope of the Arrhenius equation as shown by Equation (4):

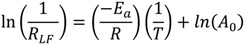
(4)
where *R_LF_* is the diameter of the low frequency impedance arc, *R* is the ideal gas constant, *T* is the temperature in Kelvin, and *A_o_* is the y-intercept. Figure 4a–c illustrate the Arrhenius plot for each data set. 

The linear data shown in Figure 4 suggests that a single rate limiting mechanism may govern the NO_x_ sensor behavior within this temperature regime and porosity range. A table of the calculated *E_a_* values is given in Table 1. The data and calculations indicated a decrease in the *E_a_* with respect to an increase in the NO concentration over a range of 5–100 ppm for each sensor. The data shown in Figure 4 and Table 1 indicated that *E_a_* increased as the porosity of the sensor increased. The increase in activation energy with respect to porosity suggests that NO_x_ and O_2_ reactions become limited possibly on account of the availability of fewer electrochemical reaction sites at the electrode/electrolyte interface. 

**Table 1:** Activation energy for various NO concentrations and sensors. Sensor A, B, and C have 50, 48, and 46% porosity, respectively.

NO (ppm)	E_a_ (kJ/mol)		
	Sensor A	Sensor B	Sensor C
**5**	109.1 ± 1.4	103.6 ± 3.9	101.8 ± 0.1
**25**	107.5 ± 1.5	102.4 ± 1.2	100.6 ± 0.1
**50**	106.8 ± 1.4	101.8 ± 1.3	99.7 ± 0.6
**100**	104.9 ± 2.1	99.8 ± 1.6	97.4 ± 0.6

Although there have been numerous studies on the effect of porosity on reaction mechanisms impacting YSZ, the experimental conditions of the reported studies are largely intended for solid oxide fuel cell applications, which makes the results less applicable to NO_x_ sensors. For porous YSZ-based gas sensors, there is evidence that reducing the porosity limits gas diffusion resulting in a slower sensor response [1]. On the other hand, increasing porosity reduces YSZ particle-to-particle contact, which decreases the TPB length and limits O^−2^ transport. As a result, charge transfer reactions as described in Equations (1) and (2) can be impeded. 

### 3.3. Oxygen Partial Pressure Dependence

An understanding of oxygen reactions at the surface and within the YSZ electrolyte can aid interpretation of NO sensing behavior as gas phase oxygen and oxygen ions participate in NO reactions. Oxygen arriving at the sensor encounters the porous YSZ electrolyte where it interacts with NO and the YSZ surface. The gas phase reaction involving O_2_, NO, and NO_2_ described in Equation (3) takes place, as well as oxygen exchange processes (*i.e.*, adsorption, dissociation, diffusion, and charge transfer). 

**Figure 5:**
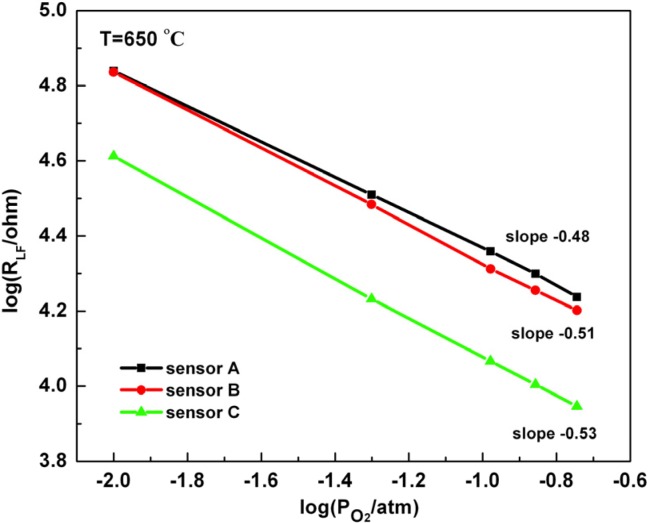
O_2_ concentration dependence of *R_LF_* for all three sensors. Sensors A, B, and C have 50, 48, and 46% porosity, respectively.

The resistance *R_LF_* associated with the low frequency impedance arc is sensitive to oxygen and can be used to interpret reaction mechanisms related to oxygen exchange processes. The power law, *R_LF_*∝ (Po_2_)*^m^*, where *m* is the slope describes this relationship, and data for each sensor is shown in Figure 5. A positive slope occurs when the fraction of occupied adsorption sites is high, and the opposite occurrence is given by the negative sign. When diffusion is rapid, which can occur in porous microstructures with a large degree of open porosity, charge transfer at the electrode/electrolyte interface can be rate limiting and *m* = ± 0.25 [10,41,42,43]. Gas diffusion is generally rate limiting when the slope is −1, which may impact to sensors with lower porosity.

In this work, the slope determined from the porous sensors was about −0.50, which has been associated with dissociative adsorption, a surface limited process [44,45,46]. Since the values are so close to −0.50, it is possible that a single process was rate limiting. Similar observations were observed by Woo *et al.*, however their reported slope was slightly higher (*m* = −0.62) suggesting that dissociative adsorption and an additional rate limiting step affected oxygen behavior [10]. 

### 3.4. YSZ Model Cluster Porosity

The atom volumes and surfaces model calculates surface areas and volumes under surfaces around atomistic structures, using the atom volumes and surfaces functionality of the Materials Studio software Visualizer. With this capability, the 40-atom YSZ model cluster volume under its van der Waals surface is calculated to be 457.41 Å^3^ using an ultra-fine grid resolution level of 0.15 Å. The volume occupied by all atoms in the YSZ cluster can be calculated as the volume of spheres whose radii are the van der Waals radii of the atoms forming the YSZ cluster, and therefore the fraction of the volume occupied by the atoms in the cluster to the total volume under the cluster van der Waals surface can be taken as an estimation of porosity of the YSZ model cluster. By doing this, the resulting porosity of the YSZ cluster used in this work is about 62% ± 2%.

**Figure 6:**
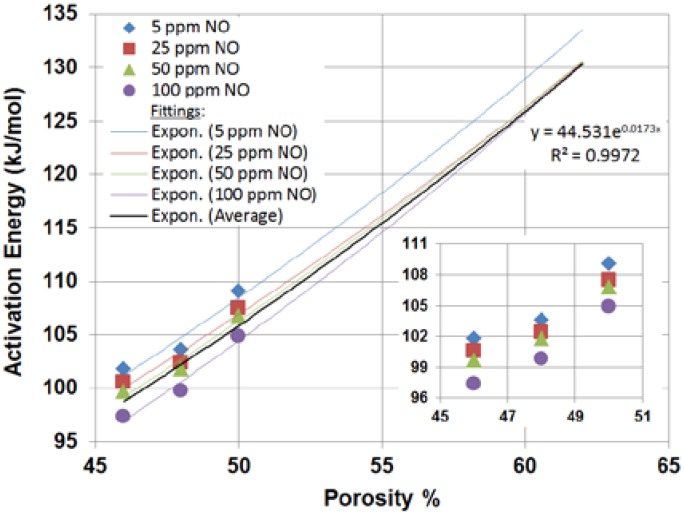
Experimental activation energies *vs.* sensor porosity with exponential fittings.

Extrapolation of the experimental data offers insight that is not possible to obtain experimentally. The firing temperatures for the sensors were limited to the range of 950–1050 °C to provide structural integrity and avoid melting the Au wire electrodes (Au melting point is 1060 °C). Although Pt is commonly used for sensor electrodes and tolerates higher firing temperatures, Pt is also a strong catalyst for O_2_ which tends to limit NO sensitivity. Experimental data of activation energy *versus* porosity (Figure 6) can be extrapolated to predict the activation energy at the 62% of porosity of the YSZ model cluster. 

In order to qualitatively compare with experimental values different fitting curves were considered and found that the one that best fit the data is an exponential function. It was observed that the exponential fitting curves corresponding to the 25, 50 and 100 ppm data points all meet at 62% porosity.

Since the modeling of Equation (3) is conducted at the stoichiometry of the reaction, it is not possible to make a quantitative comparison to the experimental data, as the NO concentration is considerable in the former. It seems that, except for the lowest 5 ppm NO case, all experimental data points lead to a common limiting case of about *E_a_* = 130 kJ/mol irrespective of the increased NO concentration (Figure 6). It is reasonable to assume then that this limit persists even when the NO concentration is significant. Hence, extrapolating an average curve between the data points for the 25, 50 and 100 ppm NO cases (R^2^ = 0.9972, fitted also with an exponential function y = 44.531e^0.0173x^) for the estimated 62% porosity representing the YSZ model cluster, a predicted activation energy was obtained (Figure 6). Since the YSZ cluster porosity is based on a qualitative measure (±2%), the predicted activation energy for the YSZ cluster used for this study is in the 125.9–134.9 kJ/mol range for a 600–700 °C temperature interval.

### 3.5. Reaction Path of Equation (3)

**Figure 7:**
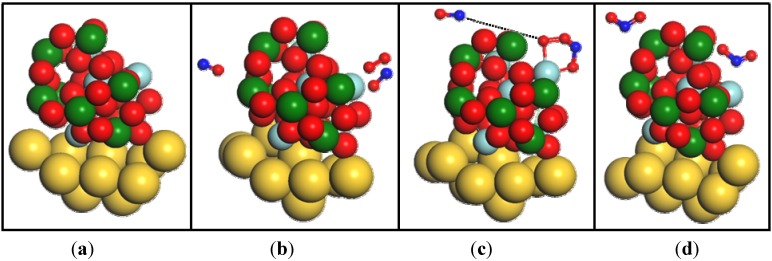
Ground state conformations of the (**a**) YSZ/Au model cluster, (**b**) reactant of Equation (3) (YSZ/Au cluster with 2NO and O_2_), (**c**) transition state structure, and (**d**) product of Equation (3) (YSZ/Au cluster with 2NO_2_ molecules). 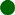: Y, 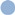: Zr, 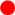: O, 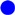: N, and 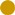: Au.

Equation (3) describes gas-phase reactions known to occur between O_2_, NO and NO_2_ at equilibrium. Based on a related study by the authors, it was verified that the experimental conditions presented here were made under equilibrium. To further interpret the relationship between Equation (3) and NO_x_ sensing, the reactivity of the YSZ/Au model cluster [Figure 7(a)] was tested at the PWC/DNP theory level by the addition of 2NO and an O_2_ molecules, according to the stoichiometry of Equation (3). A reactant [Figure 7(b)], transition state [Figure 7(c)], and product [Figure 7(d)] of Equation (3) were found after performing geometry optimizations. 

**Figure 8:**
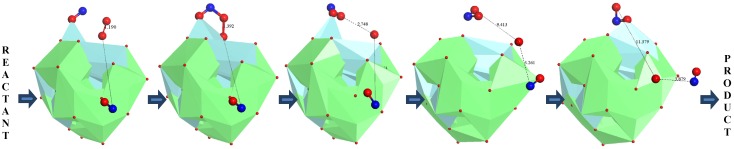
Reaction path corresponding to Equation (3) for the YSZ/Au model cluster at the PWC/DNP theory level. 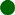: Y, 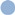: Zr, 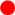: O, 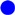: N.

The reaction path corresponding to Equation (3) for the YSZ/Au model cluster at the PWC/DNP theory level is shown in Figure 8. In this figure, the Au support cluster has been removed (as it did not play a role in this merely surface reaction), and the YSZ cluster is shown using the polyhedron atom representation to better illustrate the surface reaction that takes place.

When selected, the polyhedron atom representation indicates that solid coordination polyhedra are drawn around YSZ cluster cations, with the number of corners corresponding to the coordination number, as defined by the bonding connectivity. The path (Figure 8) shows oxygen surface reactions that begin with adsorbed NO (NO*) association with adsorbed O_2_ (O_2_*) on a Zr surface site, followed by O_2_ dissociative adsorption, atomic oxygen diffusion and migration to free NO molecules, and further NO_2_ formation.

**Figure 9:**
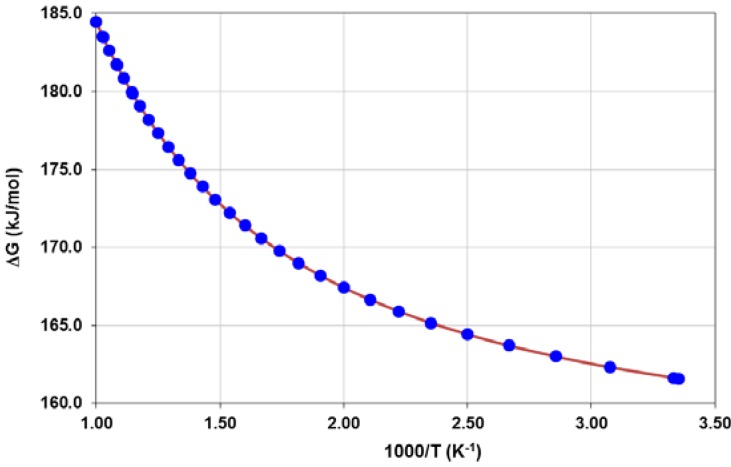
Calculated Gibbs Free energy barriers at different temperatures corresponding to the forward reaction given by Equation (3) at the PWC/DNP theory level for the YSZ/Au model cluster.

The calculated internal energy of activation at absolute zero, ΔU, and the Gibbs Free energy barrier (internal energy of activation with the thermal corrections to the Gibbs Free energy at different temperatures included), ΔG(T), for the forward reaction given by Equation (3) at the PWC/DNP theory level are ΔU = 156.2 kJ/mol and ΔG(650 °C) = 181.7 kJ/mol (averaged for the 600–700 °C temperature range of the experimental work) respectively. Gibbs Free energy barriers at diverse temperature conditions are given in Figure 9.

The calculated internal energy of activation and free energy barrier of Equation (3) seem overestimated at the PWC/DNP theory level when compared to experimental findings, as expected when LDA functionals are used [35]. In order to provide better kinetic results, the structures optimized at the PWC/DNP theory level were used to conduct single point energy calculations at the PW91/DNP, PBE/DNP, and RPBE/DNP theory levels to obtain new ΔU and ΔG values for Equation (3).

Additionally, the temperature corrections used in the calculation of ΔG in those cases are the ones calculated at the PWC/DNP theory level. Therefore, it is worth to note, that since full geometry optimizations were not performed at these three GGA levels of theory, only trends can be drawn from the results presented in Table 2.

**Table 2:** Energy obtained after TS search and calculation of internal energy of activation of Equation (3). *Single point energy calculations on structures fully optimized at the PWC/DNP theory level. The temperature corrections used in the calculation of ΔG in those cases are the ones calculated at the PWC/DNP theory level.

Equation (3)	Theory Level			
	PWC	PW91 *	PBE *	RPBE *
**∆U (kJ/mol)**	156.2	137.7	133.5	123.6
**∆G(650 °C) (kJ/mol)**	181.7	163.2	159.0	149.1

A qualitative comparison with the experimental data for the extrapolated value of the activation energy at 62% ± 2% porosity (in the 125.9–134.9 kJ/mol range) representing the YSZ model cluster, indicates that the calculated ΔG(650 °C) values are in reasonable agreement with experimental findings considering the expected error in energies of 8–20 kJ/mol [15,16,25]. The GGA methods PW91, PBE, and RPBE perform better than the LDA PWC functional, as the calculated free energy barriers are in better comparison with experimental data, particularly when the RPBE functional is used.

Making quantitative comparisons to other studies is challenging as porous YSZ electrolytes are a relatively new area of study for gas sensing, and limited research has been reported on the impact of porosity on the mechanisms governing NO_x_ sensing. Nonetheless, results of other sensor studies do indicate that NO_x_ sensitivity is strongly linked to reactions occurring on the porous YSZ surface [13]. In addition, significant interaction between NO_x_ and YSZ has been verified using temperature-programmed desorption, diffuse reflectance infrared Fourier transform spectroscopy, and chemical reactivity methods [11]. The experimental and modeling data presented here provide consistent findings as both support dissociative adsorption as a potential rate limiting mechanism; and, the activation energy values agree with the same trend where increasing activation energy corresponded with increasing porosity.

## 4. Conclusions

NO_x_ exhaust gas sensors play a critical role in monitoring diesel emissions and communicating with the vehicle diagnostic system to regulate engine operation. Sensors with greater sensitivity and accuracy are necessary to further reduce air pollution and satisfy future emission requirements. Y-stabilized ZrO_2_ is commonly used as the sensor electrolyte given its tolerance for the harsh exhaust gas environment. Recent research indicates the microstructure of YSZ plays a role in NO_x_ sensing, as sensors with porous *vs.* dense YSZ demonstrate greater sensitivity to NO_x_ under certain operating conditions. The aim of the present study was to interpret the reaction(s) governing NO_x_ sensing for porous YSZ based sensors.

The effect of porosity on sensing mechanisms in NO_x_ sensors composed of YSZ microstructures with porosities ranging from 46%–50% was studied using experimental techniques. Although the sensor porosity was limited to this range, a strong dependence on porosity was observed in the electrical response of sensors with various porosities. Temperature dependent experimental results from impedance data used to calculate activation energies indicated higher activation energies as the porosity increased. This suggested that porosity impedes the ability of certain reactions to proceed, possibly as a result of limited oxygen ion transport. Analysis of the oxygen partial pressure dependence indicated dissociative adsorption as rate limiting. Similar findings were determined from computational results that depicted NO reaction steps at the YSZ surface. 

A 56-atom YSZ/Au model cluster was built using the δ-YSZ cubic fluorite structure with molecular formula Y_4_Zr_3_O_12_ on a 16-Au cluster. The Local Density Approximation PWC functional in combination with the DNP basis set was used to investigate the gas-phase internal energy of activation and free energy barrier of the 2NO + O_2_ → 2NO_2_ reaction, using the linear synchronous transit and quadratic synchronous transit (LST/QST) calculation with conjugate gradient minimization within the Transition State search tool in DMOL^3^. The reaction path shows oxygen surface reactions that begin with adsorbed NO (NO*) association with adsorbed O_2_ (O_2_*) on a Zr surface site, followed by O_2_ dissociative adsorption, atomic oxygen diffusion and migration to free NO molecules, and further NO_2_ formation. 

The calculated internal energy of activation at absolute zero, ΔU, and the Gibbs Free energy barrier (internal energy of activation with the thermal corrections to the Gibbs Free energy at different temperatures included), ΔG(T), for the forward reaction given by Equation (3) at the PWC/DNP theory level are ΔU = 156.2 kJ/mol and ∆G(650°C) = 181.7 kJ/mol (averaged for the 600–700 °C temperature range of the experimental work) respectively. A qualitative comparison with the experimental data for the extrapolated value of the activation energy at 62% ± 2% porosity (in the 125.9–134.9 kJ/mol range) representing the YSZ model cluster, indicates that the calculated ∆G(650 °C) values are in reasonable agreement with experimental findings considering the expected error in calculated energies of 8–20 kJ/mol. The GGA methods PW91, PBE, and RPBE perform better than the LDA PWC functional, as the calculated Gibbs Free energy barriers are in better comparison with experimental data, being the RPBE functional the best one able to reproduce experimental results. 

Overall, the modeling work of NO-YSZ interactions provides an understanding of the reactions governing sensor performance. Such insight is beneficial for optimizing NO_x_ sensor microstructure, as well as interpreting and controlling sensor behavior.

## References

[1] Mukundan R., Teranishi K., Brosha E.L., Garzona F.H. (2007). Nitrogen oxide sensors based on yttria-stabilized zirconia electrolyte and oxide electrodes. Electrochem. Solid-State Lett..

[2] Park J., Yoon B.Y., Park C.O., Lee W.-J., Lee C.B. (2009). Sensing behavior and mechanism of mixed potential NO_x_ sensors using NiO, NiO(+YSZ) and CuO oxide electrodes. Sens. Actuators B.

[3] Stranzenbach M., Saruhan B. (2009). Equivalent circuit analysis on NO_x_ impedance-metric gas sensors. Sens. Actuators B.

[4] Woo L.Y., Glass R.S., Novak R.F., Visser J.H. (2011). Diesel engine dynamometer testing of impedancemetric NO_x_ sensors. Sens. Actuators B.

[5] (2010). 2008 Progress Report: Vehicle and Engine Compliance Activities, EPA-420-R-10–022.

[6] (1999). EPA’s Program for Cleaner Vehicles and Cleaner Gasoline, EPA420-F-99–051.

[7] Martin L.P., Woo L.Y., Glass R.S. (2007). Impedancemetric NO_x_ sensing using YSZ electrolyte and YSZ/Cr_2_O_3_ composite electrodes. J. Electrochem. Soc..

[8] Menil F., Coillard V., Lucat C. (2000). Critical review of nitrogen monoxide sensors for exhaust gases of lean burn engines. Sens. Actuators B.

[9] Striker T., Ramaswamy V., Armstrong E.N., Willson P.D., Wachsman E.D., Ruud J.A. (2013). Effect of nanocomposite Au–YSZ electrodes on potentiometric sensor response to NO_x_ and CO. Sens. Actuators B.

[10] Woo L.Y., Martin L.P., Glass R.S., Wang W., Sukwon J., Gorte R.J., Murray E.P., Novak R.F., Visser J.H. (2008). Effect of electrode composition and microstructure on impedancemetric nitric oxide sensors based on YSZ electrolyte. J. Am. Chem. Soc..

[11] Yang J.C., Dutta P.K. (2007). Promoting selectivity and sensitivity for a high temperature YSZ-based electrochemical total NO_x_ sensor by using a Pt-loaded zeolite Y filter. Sens. Actuators B.

[12] Mukundan R., Brosha E.L., Garzon F.H. (2003). Mixed potential hydrocarbon sensors based on a YSZ electrolyte and oxide electrodes. J. Electrochem. Soc..

[13] Woo L.Y., Glass R.S., Novak R.F., Visser J.H. (2010). Effect of electrode material and design on sensitivity and selectivity for high temperature impedancemetric NO_x_ sensors. J. Am. Chem. Soc..

[14] Peredith A., Ceder G., Wolverton C., Persson K., Mueller T. (2008). Ab initio prediction of ordered ground-state structures in ZrO_2_-Y_2_O_3_. Phys. Rev. B.

[15] Dreialer R.M., Gross E.K.U. (1990). Density Functional Theory: An. Approach to Quantum Many Body Problem.

[16] Koch W., Holthausen M.C. (2001). A Chemist’s Guide to Density Functional Theory.

[17] (2006). Materials Studio.

[18] (2003). DMOL^3^ User Guide.

[19] Amelines-Sarria O., Kolokoltsev Y., Basiuk V.A. (2010). Noncovalent 1:2 complex of Meso-tetraphenylporphine with C_60_ fullerene: A Density Functional Theory study. J. Comput. Theor. Nanosci..

[20] Basiuk V.A. (2011). Electron smearing in DFT calculations: A case study of doxorubicin interaction with single-walled carbon nanotubes. Int. J. Quantum Chem..

[21] Kolokoltsev Y., Amelines-Sarria O., Gromovoy T.Y., Basiuk V.A. (2010). Interaction of Meso-tetraphenylporphines with C_60_ fullerene: Comparison of several Density Functional Theory functionals implemented in DMol^3^ Module. J. Comput. Theor. Nanosci..

[22] Famulari A., Raos G., Baggioli A., Casalegno M., Po R., Meille S.V. (2012). A solid state Density Functional study of crystalline thiophene-based oligomers and polymers. J. Phys. Chem. B.

[23] Melis C., Colombo L., Mattoni A. (2011). Self-assembling of poly(3-hexylthiophene). J. Phys. Chem. C.

[24] Yu G., Yin S., Liu Y., Shuai Z., Zhu D. (2003). Structures, electronic states, and electroluminescent properties of a Zinc(II) 2-(2-Hydroxyphenyl)benzothiazolate complex. J. Am. Chem. Soc..

[25] Becke A.D. (1993). Density-functional thermochemistry III. The role of exact exchange. J. Chem. Phys..

[26] Govind N., Petersen M., Fitzgerald G., King-Smith D., Andzelm J. (2003). A generalized synchronous transit method for transition state location. Comput. Mater. Sci..

[27] Grillo M.E., Govind N., Fitzgerald G., Stark K.B. (2004). Computational material science with materials studio: Applications in catalysis. Lect. Notes Phys..

[28] Dathara G.K.P., Mainardi D.S. (2010). Kinetics of hydrogen desorption in NaAlH_4_ and Ti-Containing NaAlH_4_. J. Phys. Chem. C.

[29] Idupulapati N.B., Mainardi D.S. (2008). A DMOL^3^ study of the methanol addition-elimination oxidation mechanism by methanol dehydrogenase enzyme. Mol. Simul..

[30] Idupulapati N.B., Mainardi D.S. (2009). Coordination and binding of ions in Ca^2+^- and Ba^2+^-containing methanol dehydrogenase and interactions with methanol. J. Mol. Struct. THEOCHEM.

[31] Idupulapati N.B., Mainardi D.S. (2010). Quantum chemical modeling of methanol oxidation mechanisms by methanol dehydrogenase enzyme: Effect of substitution of calcium by barium in the active site. J. Phys. Chem. A.

[32] Hill C.G. (1977). An Introduction to Chemical Engineering Kinetics & Reactor Design.

[33] Ramachandran B., Peterson K.A. (2003). Potential energy surfaces for the 3A and 3A electronic states of the O(^3^*P*) + HCl system. J. Chem. Phys..

[34] Xie T., Bowman J.M., Ramachandran B., Peterson K.A. (2003). Quantum calculations of the rate constant for the O(^3^*P*) + HCl reaction on new Ab Initio 3A and 3A surfaces. J. Chem. Phys..

[35] Jones R.O., Gunnarsson O. (1989). The Density Functional formalism, its applications and prospects. Rev. Mod. Phys..

[36] Perdew J.P., Chevary J.A., Vosko S.H., Jackson K.A., Pederson M.R., Singh D.J., Fiolhais C. (1992). Atoms, molecules, solids, and surfaces: Applications of the generalized gradient approximation for exchange and correlation. Phys. Rev. B.

[37] Perdew J.P., Burke K., Ernzerhof M. (1996). Generalized gradient approximation made simple. Phys. Rev. Lett..

[38] Hammer B., Hansen L.B., Norskov J.K. (1999). Improved adsorption energetics within Density-Functional Theory using revised Perdew-Burke-Ernzerhof functionals. Phys. Rev. B.

[39] Macdonald J.R. (1987). Impedance Spectroscopy: Emphasizing Solid Materials and Systems.

[40] Steil M.C., Thevenot F. (1997). Desensification of yttria-stabilized zirconia. J. Electrochem. Soc..

[41] Takeda Y., Kanno R., Noda M., Tomida Y., Yamamoto O. (1987). Cathodic polarization phenomena of perovskite oxide electrodes with stabilized zirconia. J. Electrochem. Soc..

[42] Vayenas C.G., Bebelis S.I., Yentekakis I.V., Neophytides S.N. (1997). CRC Handbook.

[43] Wang D.Y., Nowick A.S. (1979). Cathodic and anodic polarization phenomena at platinum electrodes with doped CeO_2_ as electrolyte: I. steady-state overpotential. J. Electrochem. Soc..

[44] Murray E.P., Tsai T., Barnett S.A. (1998). Oxygen transfer processes in (La,Sr)MnO_3_/Y_2_O_3_- stabilized ZrO_2_ cathodes: An impedance spectroscopy study. Solid State Ionics.

[45] Koyama M., Wen C., Masuyama T., Otomo J., Fukunaga H., Yamada K., Euguchi K., Takahashi H. (2001). The mechanism of porous Sm_0.5_Sr_0.5_CoO_3_ cathodes used in solid oxide fuel cells. J. Electrochem. Soc..

[46] Van Herle J., McEvoy A.J., Thampi K.R. (1996). A study on the La_1−x_Sr_x_MnO_3_ oxygen cathode. Electrochim. Acta.

